# Music-Based Cognitive Remediation Therapy for Patients with Traumatic Brain Injury

**DOI:** 10.3389/fneur.2014.00034

**Published:** 2014-03-24

**Authors:** Shantala Hegde

**Affiliations:** ^1^Cognitive Psychology and Cognitive Neurosciences Laboratory, Department of Clinical Psychology, Neurobiology Research Center, National Institute of Mental Health and Neuro Sciences (NIMHANS), Bangalore, India

**Keywords:** traumatic brain injury, cognitive rehabilitation, neurologic music therapy, neuromusicology, music cognition, music therapy

## Abstract

Traumatic brain injury (TBI) is one of the common causes of disability in physical, psychological, and social domains of functioning leading to poor quality of life. TBI leads to impairment in sensory, motor, language, and emotional processing, and also in cognitive functions such as attention, information processing, executive functions, and memory. Cognitive impairment plays a central role in functional recovery in TBI. Innovative methods such as music therapy to alleviate cognitive impairments have been investigated recently. The role of music in cognitive rehabilitation is evolving, based on newer findings emerging from the fields of neuromusicology and music cognition. Research findings from these fields have contributed significantly to our understanding of music perception and cognition, and its neural underpinnings. From a neuroscientific perspective, indulging in music is considered as one of the best cognitive exercises. With “plasticity” as its veritable nature, brain engages in producing music indulging an array of cognitive functions and the product, the music, in turn permits restoration and alters brain functions. With scientific findings as its basis, “neurologic music therapy” (NMT) has been developed as a systematic treatment method to improve sensorimotor, language, and cognitive domains of functioning via music. A preliminary study examining the effect of NMT in cognitive rehabilitation has reported promising results in improving executive functions along with improvement in emotional adjustment and decreasing depression and anxiety following TBI. The potential usage of music-based cognitive rehabilitation therapy in various clinical conditions including TBI is yet to be fully explored. There is a need for systematic research studies to bridge the gap between increasing theoretical understanding of usage of music in cognitive rehabilitation and application of the same in a heterogeneous condition such as TBI.

## Traumatic Brain Injury and Its Sequelae

Traumatic brain injury (TBI), an injury to the brain from an external agent or force, is one of the leading causes of disability in multiple domains of functioning. TBI may cause transient or long lasting impairment in neurological and neuropsychological functioning (1). Injury may be primary or secondary in nature. Primary injury is the direct impact of the external agent causing injury to the brain. Secondary injury is due to disordered autoregulation or any other pathophysiological changes within the brain following the injury. Hypoxic or ischemic injuries which may be a primary or secondary injury affect recovery. Depending upon the site of injury (focal or diffuse) and severity of the injury (mild, moderate, or severe), impairment is observed in the physical, psychological, and social domains of functioning. These impairments may be transient or long lasting in nature (2–5). The TBI sequelae includes physical sequelae which may be in the form of motor dysfunctions, hemiplegia, visual impairment, auditory impairment, deficits in gait etc; psychological sequelae in the form of deficits in cognitive functions and emotional problems; social sequelae in the form of loss of family or friends, or changes in the social relationship, changes in routine activities, loss of job or loss of work-related skills, inability to acquire new skills, and financial problems. It is the psychological and social consequences of TBI that are far more disabling and burdensome to the individual, the caregivers as well as the society at large (3, 6–8). The psychological and social sequelae may be observed immediately after the injury or after lapse of time. This may or may not be in concurrence with physical disability. Physical dysfunctions are easily detectable and often not a major problem in mild injury. The severity of TBI is associated positively with severity of psychosocial functioning. Cognitive deficits such as deficits in attention, information processing, planning, decision-making, memory, language, and emotional processing have significant impact on personal and socio-occupational functioning. Cognitive deficits are considered to play a central role in TBI and contribute significantly to functional recovery (4, 9).

## Cognitive Remediation in TBI

In the past three and half decade, cognitive remediation (CR) has emerged as one of the best available treatment methods to restore cognitive functions and facilitate compensatory strategies to overcome cognitive deficits following TBI or other acquired brain injury conditions (10–15). CR is described as “procedures designed to provide patients with the behavioral repertoire needed to solve problems or to perform tasks that seem difficult or impossible” (16). The terms rehabilitation and remediation conveys specific approach to treatment, although the two terms have been interchangeably used in literature to discuss treatment strategies aiming at skills development in patients needed to perform tasks that are difficult to perform due to cognitive deficits. In a technical sense, rehabilitation involves a wide array of interventions offered by a multidisciplinary team. CR comes under the umbrella of a broader treatment approach, i.e., rehabilitation (17). Cognitive rehabilitation includes methods such as remediation, compensation, and holistic or multimodal programs (6, 18). Cognitive rehabilitation is a confluence of therapeutic activities based on brain–behavior relationships. Functional improvement is achieved by re-establishing or reinforcing previously learned adaptive patterns of behavior, facilitating improvement in cognitive functions through compensatory mechanisms and sometimes facilitating new patterns of activity through external compensatory mechanisms. The goal of cognitive rehabilitation is to help patients with cognitive deficits to adapt to their disability to improve overall functioning. The holistic approach addresses the cognitive, emotional, and other non-cognitive domains of functioning as well as addresses rehabilitation in social milieu and facilitating patient to have better understanding of one’s own reaction to the consequences of brain injury (19–22). Evidence exists to support benefits of CR in improving cognitive functions such as attention, memory aphasia, functional communication, and unilateral spatial neglect (11, 12, 23). Systematic reviews on a total of 370 intervention studies on CR in TBI and other acquired brain injury have concluded that there is substantial evidence to support benefits of CR in TBI (13, 24). Meta-analysis has shown a small treatment effect size (*d* = 0.30) to large effect size (*d* = 0.71), variable due to the design of the research studies with larger effect size observed in single group pre-test and post-test comparison studies. The outcome was influenced by moderating variables such as the specific cognitive function targeted in intervention, duration between injury to treatment onset, the type of injury, and age of patients included in the intervention studies (13). Interventions to remediate cognitive deficits have often employed either paper–pencil or computer-based tasks that would enable direct training of the cognitive function as well as metacognitive training methods (i.e., self-monitoring and self-regulation) to facilitate compensatory strategies and to facilitate generalization to real-world situations (12). The most often evaluated treatment approach in the published literature aims to directly retrain the cognitive function that is impaired via cognitive drills and exercises targeting the specific cognitive function (25). Repeated practice on carefully designed exercises is considered to facilitate recovery of the damaged neural circuits and restoration of function such as attention, memory, executive functions, etc. The tasks mediated by these circuits would then lead to a near normal or normal level of functioning as comparable to the functioning due to an intact brain without any injury (16, 26–28). A careful examination of literature on CR in acquired brain injury indicates a lack of high-quality evidenced-based research studies and fraught by lack of generalizability of improvement on cognitive functions targeted in the treatment sessions to real-life situations (23, 29–31).

## Music-Based Intervention as a Technique of CR

Many innovative methods to treat TBI sequelae have been developed over the years. Music therapy is one such method. Music-based intervention methods have shown promising results in rehabilitating movement, gait-related problems (32). It has shown positive results in reducing the levels of anxiety, depression, agitation and in inducing stable mood state. Enhanced adaptive behavior following music-based intervention has been observed even during recovery from coma and later in both adult and children population (33–39). Music-based intervention has led to improvement in speech production and sensory perceptions (40, 41). Studies suggest that use of musical intervention facilitates early responsiveness in patients which in turn foster cognitive rehabilitation in the early acute phase following TBI (39). The more recent frontier is music-based CR in various neurological and neurosurgical conditions (33, 35). The application of music in CR is although a recent endeavor, the potential of music in this area of rehabilitation was put forth much earlier (42).

The use of music as a therapeutic method has a long history, perhaps dates back to prehistoric times driven by the social science model wherein music is interpreted as a facilitator of “well-being” and enhancing emotional health (43, 44). This very nature of music to reduce stress and enhance emotional health has started receiving scientific evidence from studies examining the neurochemical changes that occur when listening to or engaged in music actively. The two important markers of stress regulated by the hypothamalic-pituitary-adrenal axis (HPA or HTPA), the beta endorphin and cortisol levels have been observed to decrease significantly with music intervention (45–47). A recent review has examined the scientific work supporting therapeutic effect of music using neurochemical changes as evidence. The authors of this review work compile the evidences in four different domains viz., (a) reward, motivation, and pleasure mediated by dopamine and opioids; (b) stress and arousal mediated by cortisol, corticotropin-releasing hormone (CRH), and adrenocorticotropic hormone (ACTH); (c) immunity mediated by serotonin and the peptide derivatives of proopiomelanocortin (POMC), alpha-melanocyte-stimulating hormone and beta-endorphin; (d) social affiliation mediated by oxytocin (48). It is also hypothesized that listening to music facilitates neurogenesis or the regeneration and repair of cerebral nerves by adjusting the secretion of steroid hormones, finally leading to neural plasticity (49). Intensely pleasurable music or anticipation of a peak emotional experience whilst listening to music is known to engage the very same areas involved in other real-life emotions such as mesolimbic, the reward area of the brain, the nucleus accumbens, and an increase in dopamine levels (50–53). In fact evidence-based approach to understanding the benefits of music, especially in the area of neurological rehabilitation has been a recent endeavor (54, 55). The neurochemical marker of neural plasticity, viz., brain-derived neurotrophic factor (BDNF) as a marker of cognitive improvement via music-based intervention is an area of investigation yet to be carried out. Advancement in research techniques and tools to study neural correlates of cognitive processes and functions such as EEG/ERP, fMRI, TMS, rTMS, etc has brought newer insights into the underlying process and therapeutic benefits of music-based intervention. A paradigm shift in the field of music therapy has been significantly influenced by the growth in the areas of neuromusicology, music cognition, and neurochemistry of musical process. The shift in music therapy from a social science and interpretive models to neuroscientific models has been set forth (44).

In the field of neuroscience today, music is considered as a powerful tool to understand brain functions and music–brain–behavior interactions. Music engages a host of cognitive processes such as acoustic analysis, information processing, sensory motor integration, learning, memory, decision making, emotion, and creativity. The neuroscientific models of music therapy is based on the principle that, “plasticity” as its veritable nature, brain engages in producing music by indulging an array of cognitive functions and in turn gets restored and altered via music even in the non-musical domains of functioning. In other words, music can stimulate complex cognitive, affective, and sensorimotor processes in the brain that can be generalized and transferred to non-musical therapeutic applications (56–61).

**Figure 1:**
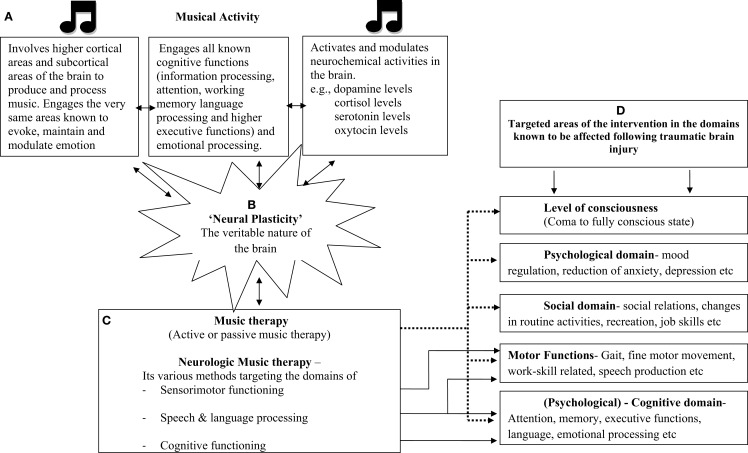
**Schematic representation of the effect of music on neural, cognitive, neurochemical functions, and how music therapy which involves musical-based activities (active and passive) has effect on various domains of functions known to be affected following traumatic brain injury**. Few key references under each section: **(A)** (46, 48, 49, 58, 60, 85); **(B)** (57, 70, 71, 86–88); **(C)** (43, 44); **(D)** (33, 34, 36, 39, 76, 89, 90)

Scientific evidence linking music and cognitive functions is more than impressive, thanks to the findings from the emerging field of music cognition and neuromusicology. Indulging in music is considered as an exercise of cognitive flexibility. Creatively working with various dynamic features of music such as pitch and rhythm is known to involve attentional networks and executive functions. Since music is known to involve all known cognitive processes, as a biological phenomenon, it is viewed as an “honest signal” for cognitive and emotional flexibility and fitness (62). Temporal cues in music and rhythm engage not only the motor system but play a crucial role in arousal, orientation, and sustenance of attention. Rhythmic patterns synchronize with the internal oscillators in accordance with its temporal regularity, thereby having its effect on attentional processes (63–66). Similarly, music provides a temporal–metrical structure that facilitates perceptual grouping and chunking of the information being processed or learnt as well as can be used as a mnemonic device in memory formation. Listening to polyphonic music has shown to engage neural circuits underlying multiple forms of working memory, attention, semantic processing, target detection, and motor imagery, in turn indicating that music listening engages brain areas that are involved in general functions rather than music-specific areas (67). Music also engages all limbic and paralimbic brain areas, which are crucial in evoking, maintaining, and modulating emotion (51, 68, 69). It seems plausible that engaging in music would not only stimulate the various centers of the brain including the emotion areas but music can also be systematically used in altering and regulating the cognitive processes involved, which can be further generalized to non-musical domains of functioning. So much so, musicians are considered as the best model to study neural plasticity due to the effects of intense training involving sensorimotor training such as music (70, 71). A schematic representation of the effect of music on the functioning of the brain and the various domains of functioning affected in TBI addressed by music therapy is presented as Figure 1.

## Neurologic Music Therapy

Findings from the field of neuroscience have provided an edifice leading to the development of a science-based approach to music therapy practice and research. This has been termed as neurologic music therapy (NMT). NMT is defined as “the therapeutic application of music to cognitive, sensory, and motor dysfunctions due to neurologic disease of the human nervous system” (43, 44, 72). NMT is based on the “rational scientific mediation model” (RSMM). This dynamic model was conceptualized to develop a systematic epistemology for translational research, linking scientific findings in neurological, psychological, and physiological foundations of music cognition and production to rehabilitation of functions in the non-music domain. NMT techniques are standardized in its terminology and application, thereby lending itself for systematic research studies. Standardized techniques and application procedures had eluded music therapy in the past (43, 44, 56). NMT techniques, hitherto have been designed to address three areas of functioning, viz.,

- Sensorimotor functioning: aims at rehabilitating motor functions, mobility, strength, endurance, cadence, and co-ordination of gross and fine motor movements in lower and upper extremities (43, 73).
- Speech and language functioning: aims at rehabilitating vocal control, speech production, and meaningful usage of verbal and non-verbal symbols in communication (43, 74).
- Cognitive functioning: aims at rehabilitating basic and higher order cognitive functions such as attention, memory, executive functions, and psychosocial skills (43, 72, 75). A brief overview on the specific techniques developed toward remediation of cognitive functions is listed in Table 1.

**Table 1:** **Brief overview on the techniques of NMT under each of the cognitive domain targeted for intervention**.

Domain of cognitive function	Technique	A brief description
Attention and perception training	Musical sensory orientation training (MSOT)	This technique targets sensory stimulation, arousal orientation, vigilance, and sustained attention using live or recorded music. This may also involve engaging the patient in simple musical exercises to target sustained attention
	Musical neglect training (MNT); auditory perception training (APT)	This technique includes both active musical exercises and passive listening. The active exercises chiefly are structured in spatial configurations to enable training of visual attention in the neglected area. The exercises are specifically structured in time, tempo, and rhythm to enable patients to focus repeatedly on the neglected visual field
	Musical attention control training (MACT)	This technique includes both active musical exercises and listening tasks. Musical stimuli are both composed of material and exercises that demand improvisation. Musical elements are organized to trigger different musical responses by engaging focused, sustained, selective, or divided attention
Memory	Musical mnemonics training (MMT)	This technique targets memory encoding and retrieval functions. Includes musical exercises of recalling sounds or lyrics such as songs, rhymes, or chants
	Associate mood and memory training (AMMT)	This technique focuses on three aspects – to facilitate memory recall by inducing mood-congruent state; to facilitate memory recall by accessing associated mood and memory network via music; to enhance memory formation by inducing positive emotional state
Executive functions	Musical executive function training (MEFT)	This technique is administered in either individual or group sessions. Includes exercises of musical composition and improvisation targeting executive functions such as working memory, problem solving, reasoning, decision-making, etc.
Psychosocial skills	Psychosocial behavior training; music psychotherapy counseling (MPC)	This technique includes guided music listening, musical role playing, and exercises of composing and improvising music. The exercises are used to target mood regulation, emotional expression, cognitive coherence, and reality orientation. The goal is to improve the functions in order to facilitate social interaction and overall psychosocial functioning

*For detailed description of each of the techniques, kindly refer to Ref. (43, 75)*.

The CR techniques via NMT is also based on the “transformational design model” (TDM) that brings together traditional cognitive rehabilitation approaches with music therapy approaches. First of all, CR via NMT begins with the first of a systematic neuropsychological evaluation to outline the specific area that requires therapeutic attention. Following this, the therapeutic goals and objectives are set. Then, the non-musical exercises in the traditional CR approach are enriched with the techniques of NMT. The final goal is to facilitate transference of improved cognitive function to non-musical domains of functioning and everyday functioning. This is targeted by providing home-work exercises with the aim of enabling generalization to everyday situation (44, 75).

Neurologic music therapy has shown promising results in remediation of motor functioning such as arm and gait control in patients suffering from stroke, Parkinson’s disease, cerebral palsy (76–78), and speech and language rehabilitation in various neurological conditions (40, 79, 80). CR is the most recent domain to be addressed via NMT. The systematic approach in this direction has spurred from the evidence that music and rhythm engage not just the motor system but also cognitive functions. The present paper will limit its focus on the third area of functioning, the cognitive functioning specific to its application TBI.

## Music-Based Cognitive Remediation in TBI, the Journey So Far

Despite the strong evidence linking music and its effect on cognition and emotion, by far, scientific evidence for effectiveness of music as to improve cognition in TBI is surprisingly weak with only a few studies and no randomized controlled trials. A review on music therapy literature indicates that only a handful of research have examined music therapy for CR in neurological conditions and far lesser studies carried out in TBI (33, 81–83). A systematic review on music therapy in acquired brain injury examined seven studies which were either randomized or quasi-randomized controlled trials with a total of 184 participants showed that rhythmic auditory stimulation (RAS) was effective in improving gait parameters including gait velocity, stride strength, cadence, and gait symmetry in acquired brain injury conditions. This review study could not comment on the effect of music therapy on other outcomes such as upper extremity motor function, speech, pain perception, and behavioral (agitation) and cognitive orientation due to insufficient data (84). This meta-analytic study examining effectiveness of music therapy for acquired brain-injured conditions found only a handful of randomized controlled group studies and none of the studies included in the final data analysis examined CR in TBI (84). So far, there has been only one study examining remediation of executive functions in TBI using music therapy. This preliminary study using a quasi experimental design examined the immediate effect of NMT in a group-setting on patients with brain injury. Of the treatment group included (*n* = 31), 77.42% of patients had TBI and the remaining sample were patients with cerebrovascular accident (12.90%), seizure disorder (6.45%), and brain tumor (3.23%). The treatment group received four brief sessions of NMT lasting for 30 min duration and each session targeted one of the following functions: attention, memory, executive functions, and emotional adjustment. The control group patients (*n* = 23) received rest period for the same duration in a quiet room for a period of 4 days. The control group participants comprised of patients with TBI (86.95% of the sample), cerebrovascular accident (4.35%), and toxic exposure (8.70%). The study showed that NMT was effective in bringing about significant positive changes in the domains of executive functions, mental flexibility to be specific with a large effect size (*d* = 1.21), and significant decrease in depressed mood with a medium effect size (*d* = 0.52) and anxiety although with a small effect size (*d* = 0.28). This brief single session intervention did not bring about significant changes in attention and memory (33). The study did not examine sustenance of improved functions over time. The findings emphasize the need for longer duration of intervention especially for cognitive functions such as attention and memory.

## Conclusion

Outcome research on music-based CR in TBI is in its infancy. There is a gap between theoretical understanding of potential role of music as a CR method and systematic evaluation of its efficacy in TBI patients. With exponential growth in the scientific evidence in linking music and cognitive functions, the gap in application of music in CR in TBI would definitely be a temporary phenomenon. So far, the set-back in carrying out such research studies may be due to the very abstract nature of music and limited understanding of the link between music and specific cognitive functions. A clearer understanding of music engaging a host of cognitive process has been possible over the past two to three decades with the emergence of scientific evidence from the fields of neuromusicology and music cognition. Until this time, there was an overpowering view of music as a therapeutic method for enhancing well-being with greater emphasis on emotional components in therapy. In addition, the difficulty may have been due to the very heterogeneous nature of TBI with varied cognitive profile. NMT methods have been successful in designing methods and techniques to address specific cognitive functions, even by circumventing the emotional component of music in the therapeutic setting. Additional markers such as neurochemical changes following NMT, such as BDNF or functional changes observed using methods such as fMRI, EEG/ERP would add to the scientific strength of the future investigations. It would not be too long before the effectiveness of NMT methods to improve cognitive functions is systematically evaluated in TBI as well as other neurological and neurosurgical conditions. A ubiquitous phenomenon like music and systematic techniques such the NMT can be main-stream holistic treatment method in TBI and other clinical conditions.

## Conflict of Interest Statement

The author declares that the research was conducted in the absence of any commercial or financial relationships that could be construed as a potential conflict of interest.
